# Retrograde versus Antegrade Approach for the Management of Large Proximal Ureteral Stones

**DOI:** 10.1155/2016/6521461

**Published:** 2016-09-27

**Authors:** Stavros Sfoungaristos, Ioannis Mykoniatis, Ayman Isid, Ofer N. Gofrit, Shilo Rosenberg, Guy Hidas, Ezekiel H. Landau, Dov Pode, Mordechai Duvdevani

**Affiliations:** ^1^Hadassah and Hebrew University Medical Center, Department of Urology, Jerusalem, Israel; ^2^G. Gennimatas Hospital, 1st Department of Urology, Aristotle University, Thessaloniki, Greece

## Abstract

*Objective*. To evaluate and compare the efficacy and safety of retrograde versus antegrade ureteroscopic lithotripsy for the treatment of large proximal ureteral stones.* Patients and Methods*. We retrospectively analyzed the medical records of patients with proximal ureteral stones >15 mm, treated in our institution from January 2011 to January 2016. Intraoperative parameters, postoperative outcomes, and complications were recorded and compared between the two techniques.* Results*. Our analysis included 57 patients. Thirty-four patients (59.6%) underwent retrograde and 23 patients (40.4%) underwent antegrade ureteroscopy. There was no significant difference in patients' demographics and stone characteristics between the groups. Stone-free rate was significantly higher (*p* = 0.033) in the antegrade group (100%) compared to retrograde one (82.4%). Fluoroscopy time, procedure duration, and length of hospitalization were significantly (*p* < 0.001) lower in retrograde approach. On the other hand, the need for postoperative stenting was significantly lower in the antegrade group (*p* < 0.001). No difference was found between the groups (*p* = 0.745) regarding postoperative complications.* Conclusions*. Antegrade ureteroscopy is an efficient and safe option for the management of large proximal ureteral stones. It may achieve high stone-free rates compared to retrograde ureteroscopy with the drawback of longer operative time, fluoroscopy time, and length of hospitalization.

## 1. Introduction

Current treatment of large proximal ureteral stones poses a therapeutic challenge and the best treatment modality still remains controversial [1]. Although extracorporeal shock wave lithotripsy (SWL) represents a valuable option, retrograde ureteroscopic lithotripsy (URSL) and antegrade URSL are both recommended as first-line treatment option according to most recent guidelines [1]. However, there is no clear consensus in the literature regarding the optimal option. Large stone burden, concomitant impaction, and stone location in the proximal ureter are all factors that may decrease postoperative stone-free rate of retrograde approach and may lead to stone retropulsion into the kidney. On the other hand, antegrade technique is considered more invasive.

The aim of the present study is to analyze several clinical, intraoperative, and postoperative factors and to compare the efficacy of retrograde versus antegrade URSL for the treatment of large proximal ureteral stones >1.5 cm. Furthermore, we evaluate the rate and grade of postoperative complications.

## 2. Patients and Methods

After we obtained an approval by Ethics Committee of our institution, we conducted a retrospective analysis of a prospectively collected database including the medical records of patients who underwent antegrade or retrograde ureteroscopy for large proximal ureteral stones >15 mm between January 2011 and January 2016. All procedures were performed by a single, fellowship-trained surgeon. Inclusion criteria were age >18 and the presence of a stone >15 mm located in the proximal ureter. Proximal ureter was defined as the part of ureter between ureteropelvic junction and the upper part of the sacroiliac joint. Patients with anatomical anomalies were excluded. A preoperative noncontrast computed tomography scan was available in all patients through Picture Archiving and Communication System of our hospital network. Several clinical and imaging parameters were collected and evaluated. Stone burden was calculated in square millimeters, using the ellipsoid formula [2]: length*∗*width*∗π∗*0.25, where *π* = 3.14. Stone density was measured in Hounsfield units.

Antegrade ureteroscopy was performed in prone position. Following the insertion of a ureteral catheter, opacification of the collecting system and intraoperative fluoroscopy were utilized to identify the optimal calyx for puncturing. A guidewire was inserted through the collecting system and sent down into the ureter. The guidewire was exchanged with a 0.038 in extrastiff wire. Balloon dilatation was used to create a 30 F tract and a 15.5 F flexible nephroscope (Karl Storz GmbH & Co., Tuttlingen, Germany) was utilized to identify the stone. Fragmentation was done with 365 *μ*m Ho:YAG laser fiber and Medilas H Solvo laser generator (Dornier MedTech GmbH, Germany). Remaining fragments were removed with 2.2 F tipless stone basket. Following complete stone removal, a 26 F rigid nephroscope was inserted for inspection of the collecting system and inspection of the operating tract while the sheath was retracted. Procedure was completed after the placement of 18 F Council catheter for drainage of the kidney. A nephrotomogram was performed the 2nd postoperative day, Council catheter was removed, and the patient was discharged.

Retrograde ureteroscopy was performed with the patient in lithotomy position. The procedure was initiated with cystoscopy and catheterization of the ipsilateral ureter with a 0.035 stiff-type, straight guidewire (safety wire) under fluoroscopic guidance. No access sheath was used in the present series. A 7 F semirigid ureteroscope (Karl Storz GmbH & Co., Tuttlingen, Germany) was used for ureteroscopy with continuous irrigation system and stone fragmentation was obtained by 550 *μ*m laser fiber. Stone fragments were extracted by a 2.2 F nitinol basket. The Storz® 7.5/8.4 Fr Flex-X^2^ (Karl Storz GmbH & Co., Tuttlingen, Germany) flexible ureteroscope was utilized for fragmentation in case of stone migration in the kidney. A double J stent or a ureteral catheter was used for ureteral stenting according to surgeon's discretion. Patients were discharged the 1st postoperative day.

Complications were categorized based on modified Clavien system [3, 4]. Stone-free outcomes were evaluated 4–6 weeks after the surgery at outpatient clinic with noncontrast computed tomography scan. Stone-free status was defined as the complete absence of residual fragments.

Statistical analysis was performed by SPSS software version 17 (SPSS Inc., Chicago, USA). Numerical variables are presented as mean ± standard deviation. Categorical variables are described by their absolute number and percent frequency. Mann-Whitney* U* test was used to compare means of numerical variables. Chi-square *χ* ^2^ test was used to compare categorical variables. All *p* values were two-tailed, with statistical significance set at 0.05.

## 3. Results

Study cohort consisted of 57 patients. Twenty-three patients (40.4%) underwent antegrade and 34 patients (59.6%) underwent retrograde ureteroscopy. Patients' demographics and stone characteristics are seen in Table 1.

Intraoperative and postoperative findings are described in Table 2. Stone-free rate was significantly higher in the antegrade URSL group (*p* = 0.033). All patients in the antegrade URSL group were stone-free after the procedure compared to 82.4% stone-free rate obtained in the retrograde group. Most of the observed complications were of grade I. No statistical difference was found in complications rate between the 2 groups (*p* = 0.745). Stone retropulsion was observed in 6 (17.6%) patients of the retrograde group while stone was not reachable in 2 (5.88%) patients. Fluoroscopy time (*p* < 0.001), procedure duration (*p* < 0.001), and length of hospitalization (*p* < 0.001) were significantly lower in retrograde approach. On the other hand, the need for postoperative stenting was lower in the antegrade one (*p* < 0.001).

## 4. Discussion

Choosing the optimal treatment modality for large proximal ureteral stones is challenging and it depends on several parameters [5]. Both antegrade and retrograde approaches are well-established techniques and both can be considered as therapeutic options. Improvements in endoscopic equipment, such as lithotripters and fiber optics, in conjunction with constantly improving surgical skills and experience have increased the efficiency and safety of minimal-invasive procedures for proximal ureteral stones management. Nowadays, antegrade and retrograde URSL are considered the primary treatment options for patients with large upper ureteral stones, supplanting the utilization of SWL [1].

There is an ongoing research interest regarding the efficiency and safety of retrograde URSL and antegrade URSL for the management of large proximal ureteral stones. Retrograde URSL is a well-established treatment option as it has been reported in several contemporary studies. Stone-free rates may reach up to 87% [6–8]. Retrograde approach combines efficacy and safety. The main advantage, compared to antegrade approach, is the usage of natural orifices and pathways minimizing, at least on a theoretical base, the risk for main complications. Establishing an artificial operative pathway is not needed and the grade of injury is less, preserving the minimal-invasive character of the procedure and the high patient tolerance, even with repeated procedures [9]. Semirigid or flexible ureteroscope can be used for reaching the stone.

The actual drawback of the technique is the increased risk of stone retropulsion into the kidney. Usually, large impacted proximal ureteral stones lead to high grade hydronephrosis. Once stone is fragmented enough to become movable, water flow and increased distal pressure may push the stone back to the kidney. Identifying and fragmenting the stone within a severely dilated system may be challenging. Additionally, the use of antiretropulsive devices or the use of a basket for grabbing and entrapping the stone is usually impossible.

Another potential drawback of the retrograde approach is the technical difficulty of the procedure. Fragmenting an impacted proximal stone may be the most challenging situation in ureteroscopy. Large proximal ureteral stones may produce extended inflammation and mucosa protrusion covering the majority of stone surface. Fragmentation can be very demanding since stone limits are not clearly identified while injury of the mucosa can lead to bleeding and obscure vision.

The advent of percutaneous nephrolithotomy emerged a trend towards antegrade approach in treating proximal ureteral stones. Usually, a flexible nephroscope is the instrument of choice for stone identification and fragmentation. Due to flexible nephroscope's larger caliber, compared to flexible ureteroscope, a better vision is provided. Additionally, larger working channel favors the usage of larger laser fibers and consequently it can diminish laser time and energy and time needed for stone fragmentation.

In our opinion, the greatest advantage of antegrade URSL is the potential for combined use of flexible and rigid nephroscope. Following stone fragmentation, fragments usually return back to the renal pelvis. Rigid nephroscope and the ultrasonic lithotripter can then identify stone fragments and suck them with ease and efficacy. Antegrade approach is also offering the possibility to treat concurrent renal stones in the same session. Antegrade approach may be also considered more advantageous, compared to retrograde one, due to several technical issues. Identifying, fragmenting, and removing a ureteral stone through a large dilated system are often easier and more ergonomic compared to stone fragmentation within a small diameter ureter.

There are several reports in the literature comparing the efficacy of antegrade and retrograde URSL. Sun et al. [10] randomized 91 patients with large (>10 mm) proximal ureteral stones to undergo either antegrade URSL (47 patients) or retrograde URSL (44 patients). Antegrade approach achieved higher stone-free rate both at discharge (95.3% versus 79.5%; *p* < 0.027) and 1 month after procedure (100% versus 86.4%; *p* < 0.026). Similarly, Xiao-Jian et al. [11] found that antegrade URSL can be more efficient than retrograde URSL for treating proximal ureteral stones >15 mm. The overall stone-free rate at 1 month after the procedure was 100% for antegrade group and 89.7% for the retrograde group. Karami et al. [12] reported their experience in the management of large impacted proximal ureteral stones based on the results of a prospective randomized study. Stone-free rate was 100% in the antegrade URSL group versus 51.4% in retrograde URSL group. Moufid et al. [13] and Bozkurt et al. [14] reported also results consistent with ours. Stone-free rates for the antegrade and retrograde approach were 95.5% versus 66.7% and 97.6% versus 82.3%, respectively.

When we are counseling a patient with large proximal ureteral stones, focusing on intraoperative and postoperative complications, it seems the common sense that antegrade approach encrypts a higher risk due to greater invasiveness. However, is it reflecting the reality? We found that there was no statistically significant difference between the 2 groups. The same result was revealed when we compared the severity of complications. Similar results have been reported in several series [13]. Despite the invasiveness of establishing a percutaneous tract, irrespective of its size, and the potential risk for immediate or late hemorrhage, antegrade approach is preserving low intrarenal pressure during the procedure. The above may significantly decrease the risk for postoperative inflammatory complications and septic phenomena. Furthermore, high irrigation flow and outflow are keeping a clear visual field and consequently decreasing the potential for accidental mucosal injury and hemorrhage. On the other hand, antegrade approach is associated with longer procedure duration and length of hospitalization. Our findings are consistent with previous reports [10, 13, 14].

Intraoperative fluoroscopy is mandatory for retrograde and antegrade URSL. Fluoroscopy can be harmful for both patients and medical staff [15]. Despite the importance of this issue, there is no data in the literature relating to it. The results of our study revealed that antegrade approach had significantly longer fluoroscopy time and consequently greater radiation exposure.

Postoperative stenting was more common in retrograde approach compared to antegrade one. It is a standard step of our technique to conduct a retrograde urethrography following fragmentation and stone removal for evaluating ureteral integrity and patency. The above assists our decision either to insert or not a ureteral stent intraoperatively. Although conclusions correlated to overall cost and quality of life cannot be extracted by our study, decreasing the rate of postoperative stenting may affect both parameters in a positive manner.

Our series presented some limitations. Firstly, the present study is limited by both its retrospective nature and being conducted at a single center. Therefore, it carries all the inherent potential issues associated with such studies. However, the above limitations may be balanced by the routine use of noncontrast computed tomography scan as the standard exam for postoperative stone-free evaluation and, additionally, by the strict criteria for defining stone-free status (complete absence of residual fragments). The single-surgeon model adds another limitation with regard to reproducibility of our results. Randomized prospective series with longer follow-up are necessary to compare the effectiveness and complications of antegrade and retrograde URSL for large proximal ureteral stones. A lower complication rate and more specifically a lower rate of postoperative bleeding, compared to the ones reported in the literature, have been reported in our study. This difference may reflect the low number of patients which represents a limitation of the study.

## 5. Conclusions

Antegrade URSL is an efficient and safe technique for treating large proximal ureteral stones. It may achieve greater stone-free rates compared to retrograde URSL. However, operative duration, fluoroscopy time, and length of hospitalization are longer.

## Figures and Tables

**Table 1:** Patients' demographics and clinical data.

	Antegrade	Retrograde	*p*

Number of patients	23 (40.4)	34 (59.6)	
*Gender*			
Male	17 (73.9)	26 (76.5)	0.826^†^
Female	6 (26.1)	8 (23.5)	
*Age*	51.2 ± 13.6	51.0 ± 17.5	0.916^‡^
*Body mass index*	27.8 ± 3.49	27.3 ± 4.92	0.386^‡^
*Side*			
Right	12 (52.2)	16 (47.1)	0.705^†^
Left	11 (47.8)	18 (52.9)	
*Stone size (mm)*	21.4 ± 4.87	19.2 ± 2.22	0.241^‡^
*Stone burden (mm* ^2^)	219.4 ± 61.8	215.9 ± 36.0	0.733^‡^
*Hounsfield units*	1069.2 ± 285.6	1020.7 ± 299.9	0.649^‡^
*Preoperative stenting*			
No	15 (65.2)	26 (76.5)	0.354^†^
Yes	8 (34.8)	8 (23.5)	
*Hydronephrosis*			
<grade 2	6 (26.1)	9 (26.5)	0.974^†^
≥grade 2	17 (73.9)	25 (73.5)	

^†^Chi-square *χ* ^2^ test, ^‡^Mann-Whitney *U* test, and sd = standard deviation.

**Table 2:** Intraoperative parameters and postoperative outcomes.

	Antegrade	Retrograde	*p*
*Procedure duration (minutes)*	63.2 ± 12.0	35.3 ± 16.0	<0.001^‡*∗*^
*Fluoroscopy time (seconds)*	172.7 ± 50.6	45.6 ± 39.4	<0.001^‡*∗*^
*Postoperative stenting*			
No	19 (82.6)	9 (26.5)	<0.001^†*∗*^
Yes	4 (17.4)	25 (73.5)	
*Length of hospitalization (days)*	4.00 ± 1.83	1.65 ± 1.48	<0.001^‡*∗*^
*Postoperative complications*			
No	17 (73.9)	27 (79.4)	0.627^†^
Yes	6 (26.1)	7 (20.6)	
*Clavien-Dindo categorization*			
Grade 0	17 (73.9)	28 (82.4)	0.745^†^
Grade 1	3 (13.0)	3 (8.8)	
Grade 2	3 (13.0)	3 (8.8)	
*Postoperative stone-free*			
No	0 (0.0)	6 (17.6)	0.033^†*∗*^
Yes	23 (100.0)	28 (82.4)	

^*∗*^Statistically significant, ^†^Chi-square *χ* ^2^ test, ^‡^Mann-Whitney *U* test, and sd = standard deviation.
